# Recent Advances in Chemistry and Bioactivities of Secondary Metabolites from the Genus *Acremonium*

**DOI:** 10.3390/jof10010037

**Published:** 2024-01-03

**Authors:** Yuning Qin, Humu Lu, Xin Qi, Miaoping Lin, Chenghai Gao, Yonghong Liu, Xiaowei Luo

**Affiliations:** Guangxi Key Laboratory of Marine Drugs, Institute of Marine Drugs, Guangxi University of Chinese Medicine, Nanning 530200, China

**Keywords:** *Acremonium*, secondary metabolites, chemical structures, bioactivities

## Abstract

*Acremonium* fungi is one of the greatest and most complex genera in Hyphomycetes, comprising 130 species of marine and terrestrial sources. The past decades have witnessed substantial chemical and biological investigations on the diverse secondary metabolites from the *Acremonium* species. To date, over 600 compounds with abundant chemical types as well as a wide range of bioactivities have been obtained from this genus, attracting considerable attention from chemists and pharmacologists. This review mainly summarizes the sources, chemical structures, and biological activities of 115 recently reported new compounds from the genus *Acremonium* from December 2016 to September 2023. They are structurally classified into terpenoids (42%), peptides (29%), polyketides (20%), and others (9%), among which marine sources are predominant (68%). Notably, these compounds were primarily screened with cytotoxic, antibacterial, and anti-inflammatory activities. This paper provides insights into the exploration and utilization of bioactive compounds in this genus, both within the scientific field and pharmaceutical industry.

## 1. Introduction

Natural products and their structural analogues have historically played a vital role in the drug discovery and development process, especially for cancer and infectious diseases [1,2]. Fungi are a hyper-diverse kingdom of life, with millions of species estimated to be present worldwide, and less than 10% of which have been described taxonomically. Of all the described and undescribed fungi, only 7% have been investigated for the chemistry of secondary metabolites [3]. Meanwhile, the secondary metabolites of filamentous fungi are largely untapped, owing to the magnitude of biosynthetic gene clusters combined with the historic number of sequenced genomes [4].

The *Acremonium* fungi, belonging to the *Hypocreataceae* family, is one of the greatest and most complicated genera in Hyphomycetes [5]. It is also a common and widely distributed fungus with about 130 species. According to the ecological habits and nutritional methods of the fungus, it is mainly divided into saprophytic, plant-parasitic, and authigenic types, and it can also survive in terrestrial or marine environments [6], but also live in close association with soil [7], plants [8], sponge [9], coral [10], algae [11], and holothurian [12], etc. Phylogenetic studies showed that the sources of *Acremonium* were related to at least three kinds of ascomycete fungi, *Hypocreaceae*, *Ergotaceae*, and *Chaetomium* [13].

As of July 2016, 356 compounds, including steroids, terpenoids, meroterpenoids, polyketides, alkaloids, peptides, and miscellaneous types, have been isolated from the genus *Acremonium* [14]. These compounds displayed a wide range of biological activities comprising antimicrobial, antitumor, immunosuppressive, antioxidant, and anti-inflammatory activities [14]. Notably, a series of ascochlorin derivatives isolated from *A. sclerotigenum* in our recent study were characterized as novel potent hDHODH inhibitors for the further development of anticancer agents [10]. The diverse and bioactive secondary metabolites from *Acremonium* have continued to attract great attention from chemists and pharmacologists.

The *Acremonium* fungi are producers of structurally diverse and pharmacologically active compounds. In this review, a total of 271 secondary metabolites (Known ones were summarized in Table S1), including 115 new compounds, were recently obtained from the genus *Acremonium* from December 2016 to September 2023. Structurally, they were classified into terpenoids (124 compounds), polyketides (66 compounds), peptides (45 compounds), steroids (18 compounds), alkaloids (9 compounds), and amides (9 compounds). Among them, 101 compounds displayed a wide range of biological activities, including antimicrobial, cytotoxic, anti-inflammatory, insecticidal, and enzyme inhibition activities. This review summarizes the sources, chemical structures, and biological activities of 115 new compounds reported in the genus *Acremonium* from December 2016 to September 2023.

## 2. Secondary Metabolites

### 2.1. Terpenoids

A total of 124 terpenoids have been reported in *Acremonium* fungi within the period 2016–2023, consisting of 31 sesquiterpenoids, 15 diterpenoids, 5 triterpenoids, and 78 meroterpenoids and miscellaneous types, while 99 compounds were found to have bioactivities. Remarkably, there are 20 new sesquiterpenoids, 11 new diterpenoids, 18 new meroterpenoids, and miscellaneous types.

#### 2.1.1. Sesquiterpenoids

**Figure 1:**
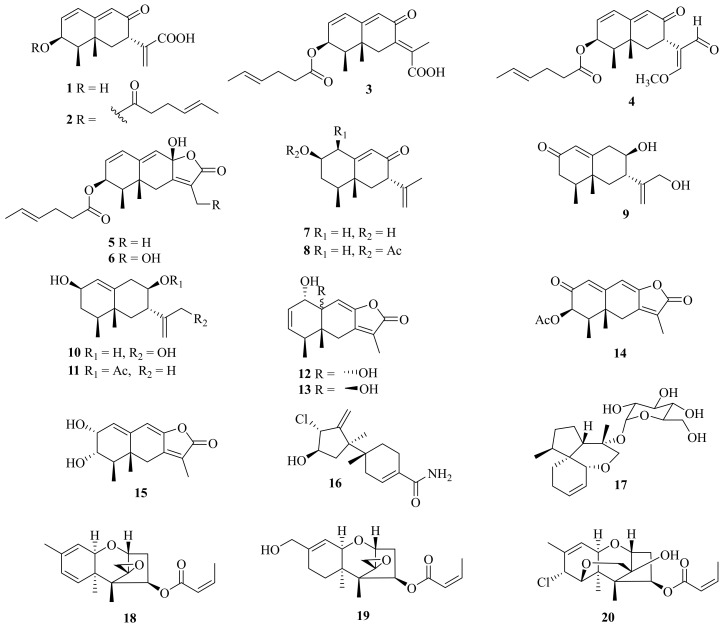
Chemical structures of sesquiterpenoids (**1**–**20**).

Several mophilane-type sesquiterpenoids, acremeremophilanes A–O (**1**–**15**), along with seven known analogues, were isolated from the deep-sea sediments derived from *Acremonium* sp. TVG-S004-0211 (Figure 1). Compounds **2**–**5** and **14** exhibited inhibition of lipopolysaccharide (LPS)-induced nitric oxide (NO) production in RAW 264.7 macrophages with IC_50_ values ranging from 8 to 45 μM [15].

One new sesquiterpenoid, marinobazzanan (**16**), was isolated from marine sediment-derived *Acremonium* sp. CNQ-049, which showed an inhibition of cancer cell migration and invasion at non-toxic concentrations of 1, 2.5, and 5 μM by down-regulating transcription factors of Snail, Slug, and Twist. In addition, marinobazzananan reduced cell motility by down-regulating the expression level of KITENIN and by up-regulating the expression level of KAI 1, and it further reduced the number of metastatic nodules in the intraperitoneal xenograft mouse model [16]. Moreover, one new acorane-type sesquiterpene glycoside, isocordycepoloside A (**17**), was isolated from the fungus *Acremonium* sp. SF-7394 [17].

Meanwhile, three new trichothecenes, including two trichothecenes, 7-dehydro-8-dehydroxytrichothecinol B (**18**) and 8-deoxy-16-hydroxytrichothecinol B (**19**), along with one trichothecene analogue, 4-((*Z*)-but-2-enoyloxy)-8-chloro-12-hydroxy-7,13-epoxytrichothec-9-ene (**20**), and four known analogues, were isolated from the fungus *A. crotocinigenum* BCC 20012. Among them, the known compound trichothecin exerted the strongest antimalarial activity against *Plasmodium falciparum* K1 with an IC_50_ value of 0.05 mg/mL, and possessed cytotoxic activity against Vero cells with an IC_50_ value of 0.13 mg/mL [18].

#### 2.1.2. Diterpenes

**Figure 2:**
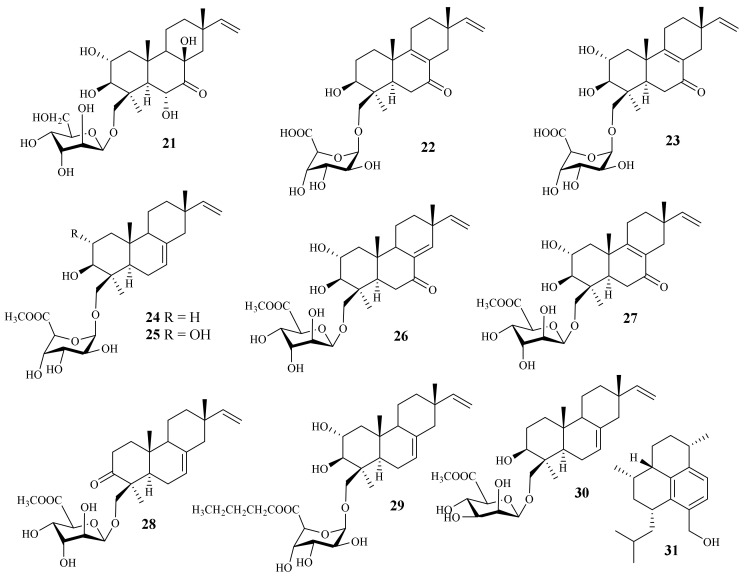
Chemical structures of sesquiterpenoids (**21**–**31**).

A chemical investigation of the marine-derived fungus *A. striatisporum* KMM 4401 resulted in the isolation of ten new diterpene glycosides, virescenosides Z9–Z18 (**21**–**30**), together with four known analogues [12] (Figure 2). One new diterpene, acrepseudoterin (**31**), was isolated from the fungus *Acremonium* sp. SF-7394. Acrepseudoterin inhibited the enzyme activity in a dose-dependent manner with an IC_50_ value of 22.8 ± 1.1 μM, which was identified as a competitive inhibitor of PTP1B [17].

#### 2.1.3. Meroterpenoids

**Figure 3:**
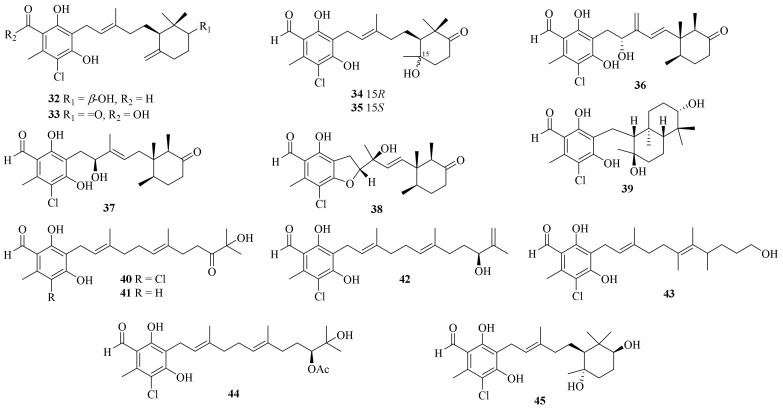
Chemical structures of meroterpenoids (**32**–**45**).

Twenty-five ascochlorin derivatives, biosynthesized through the farnesylation of orsellinic acid [19], were obtained from the coral-derived *A. sclerotigenum* GXIMD 02501, including 13 new compounds, acremochlorins A–M (**32**–**44**) (Figure 3). Compounds **32** and **44**, two novel potent human dihydroorotate dehydrogenase (hDHODH) inhibitors, induced the apoptosis of triple-negative breast cancer (TNBC) cells by up-regulating the levels of cleaved-PARP1 and cleaved-caspase7, and further effectively inhibited tumor growth in a patient-derived TNBC xenograft model without significant weight loss or obvious toxicity in mice, showing higher safety than that of brequinar [10].

Meanwhile, ascofuranone and ascochlorin, two representative ascochlorin derivatives, were also reported as potential lead candidates for drug development targeting the hDHODH of cancer cells living under a tumor microenvironment [20]. Moreover, two known potential anti-tumor ascochlorins, 3-bromoascochlorin (BAS) and ilicicolin A (Ili-A), were also obtained from the coral-derived fungus *A. sclerotigenum* GXIMD 02501. BAS could induce the apoptosis, invasion, and migration of H446 and H69AR cells, and it further suppressed the tumor growth of a small cell lung cancer xenograft mouse model by inhibiting the mitogen-activated protein kinase (MAPK)/extracellular signal-regulated kinase (ERK) pathway [21]. Moreover, Ili-A showed efficacious activity against prostate cancer cells by abrogating EZH2/AR-mediated processes and demonstrated a synergistic anti-prostate cancer effect combined with enzalutamide in vivo, revealing a novel EZH2 inhibitor for the treatment of castration-resistant prostate cancer [22].

**Figure 4:**
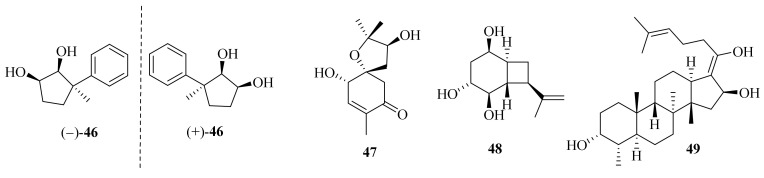
Chemical structures of miscellaneous terpenoids (**46**–**49**).

Ascofuranone and its derivatives, obtained from *A. egyptiacum*, were found as the first dual inhibitors of fumarate and oxygen respiration in *Echinococcus multilocularis* by targeting mitochondrial complexes II and III, suggesting potential lead compounds in the development of anthelminthic drugs [23]. One new ascochlorin, acremochlorin N (**45**), and a pair of new natural enantiomers, 3-phenylcyclopentane-1,2-diol (±-**46**) (Figure 4), together with nine known analogues were isolated from marine sediment-derived *A. furcatum* CS-280. All the isolates showed significant anti-*Vibrio* activities, especially against *Vibrio harveyi* and *V. alginolyticus*. Moreover, the presence of chlorine atoms in the ascochlorins could significantly enhance their antibacterial activity [24].

Meanwhile, four known ascochlorins, including ascochlorin, 10′-deoxy10′α-hydroxyascochlorin, 4′,5′-dihydro-4′-hydroxyascochlorin, and ascofuranone, were obtained from the sponge-derived *Acremonium* sp. IMB18-086. Ascochlorin and ascofuranone showed significant antibacterial activity against *Staphylococcus aureus*, methicillin-resistant *Staphylococcus aureus* (MRSA), *Bacillus subtilis*, and *Candida albicans*. Moreover, they showed significant cytotoxicity against A549 and/or HepG2 cell lines with IC_50_ values of 0.9–5.8 μM [25].

Acremine S (**47**) was isolated from the sponge *Mycale* sp. derived fungus *A. persicinum* KUFA 1007 and showed inhibitory activity against butyrylcholine esterase, which was three folds higher than that of galantamine [26]. Hexahydroacremonintriol (**48**), along with an analogue, acremonin A glucoside, were obtained from a tropical sinkhole derived from *A. masseei* CICY026. Both displayed insecticidal activity against *Myzus persicae* and/or *Rhopalosiphum padi* with settling inhibition ranging from 48% to 67% [27]. One new fusidic acid derivative, acremonidiol A (**49**), and three known analogs were obtained from the endophytic fungus *A. pilosum F47*. Among these, fusidic acid displayed a strong inhibitory effect on Gram-positive bacterium *S. aureus*, and the acetylation of the hydroxyl group at C-16 was crucial for antibacterial activity [28].

### 2.2. Peptides

A total of 45 peptides have been reported from *Acremonium* fungi during the period 2016–2023, including 33 new compounds, while 19 bioactive compounds were found.

#### 2.2.1. Linear Peptides

**Figure 5:**
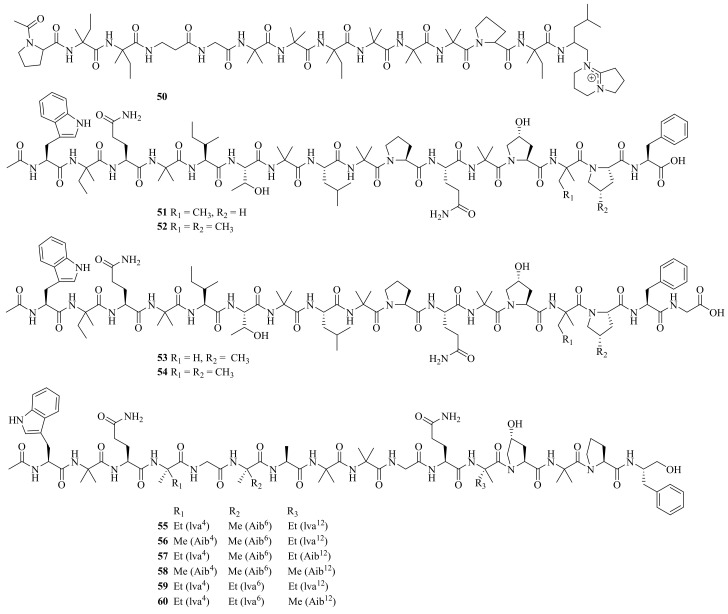
Chemical structures of linear peptides (**50**–**60**).

One new linear peptide, acremopeptin (**50**), and a known one, adenopeptin, were obtained from the soil-derived fungus *Acremonium* sp. PF1450 [29]. Moreover, four new peptaibiotics, acremotins A–D (**51**–**54**) (Figure 5), along with a known peptaibiotic XR586 were isolated from the soil-derived fungus *A. persicinum* SC0105. Acremotins A–D showed strong inhibitory activity against Gram-positive bacteria, while the MIC values of acremotin D against *S. aureus* and MRSA were 12.5 and 6.25 μg/mL, respectively. Moreover, acremotins A–D and XR586 also showed cytotoxicity against three human cancer cell lines (A549, HeLa, and HepG2), with IC_50_ values ranging from 1.2 to 21.6 μM [30].

Six new 16-residue peptaibols, acremopeptaibols A–F (**55**–**60**), along with PF1171A, were isolated from the cultures of the sponge-associated fungus *Acremonium* sp. IMB18-086. Compounds **55** and **59** showed significant antibacterial activity against *S. aureus*, MRSA, *B. subtilis*, and *C. albicans*, with MIC values ranging from 16 to 64 μM [25]

**Figure 6:**
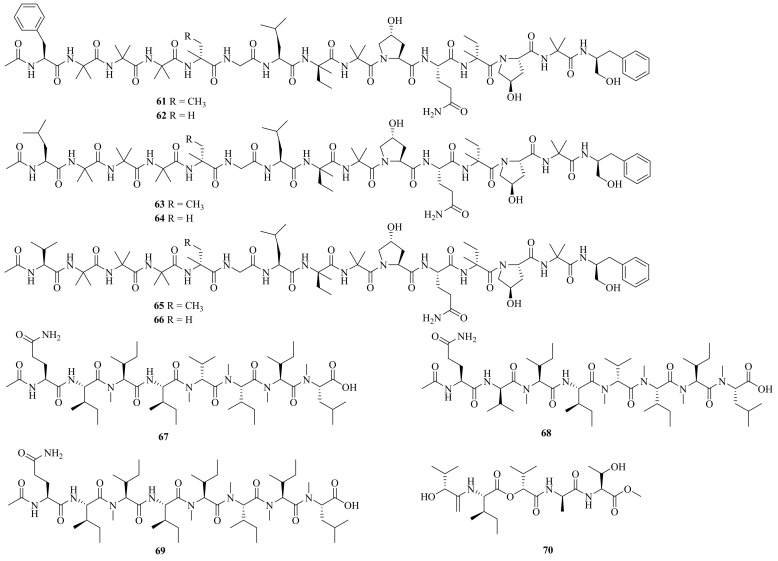
Chemical structures of linear peptides (**61**–**70**).

Six new linear pentadecapeptides, emerimicins V–X (**61**–**66**) (Figure 6), were obtained from the soil-derived fungus *A. tubakii* MT053262. Emerimicins V (**61**) and VI (**62**) displayed strong toxicity toward Zebrafish embryos. In addition, emerimicin V showed certain activity against *Enterococcus faecalis*, MRSA, and vancomycin-resistant *Enterococcus faecium* with MIC values of 64, 32, and 64 μg/mL, respectively [31].

Four new peptides, acrepeptins A–D (**67**–**70**), and three known analogs, destruxin B, guangomide A, and guangomide B, were obtained from a marine algicolous fungus *Acremonium* sp. NTU492. Acrepeptins A (**67**) and B (**68**) exhibited significant inhibitory activity on NO production in LPS-activated microglia BV-2 cells, with IC_50_ values of 12.0 ± 2.3 and 10.6 ± 4.0 mM, respectively [32].

#### 2.2.2. Cyclic Peptides

**Figure 7:**
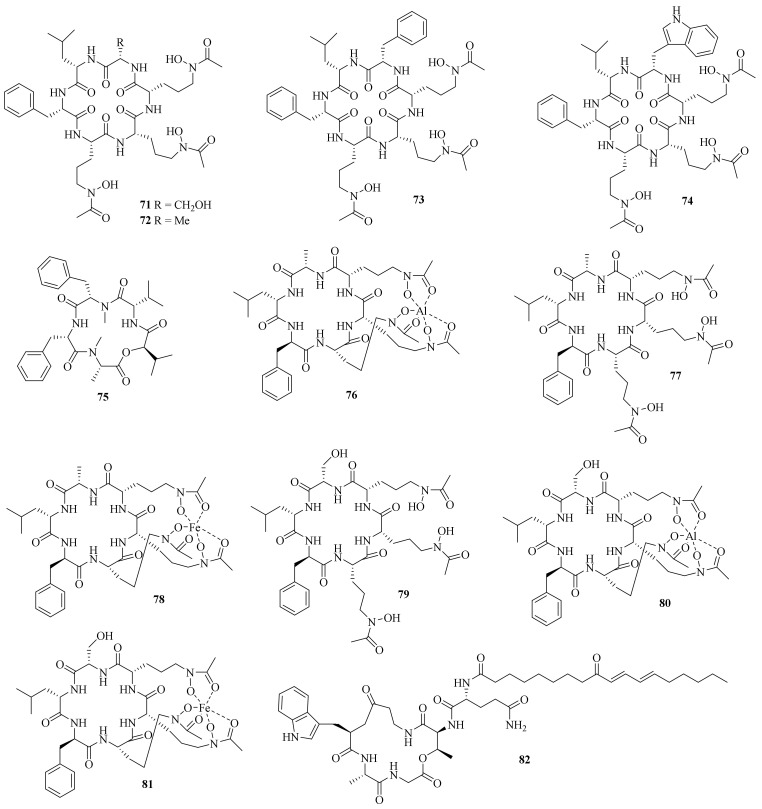
Chemical structures of cyclic peptides (**71**–**82**).

Four new hydroxamate-containing cyclopeptides, acremonpeptides A–D (**71**–**74**), together with a known one, Al (III)-acremonpeptide D, were obtained from the marine fungus *A. persicinum* SCSIO 115. Compounds **71**, **72**, and Al (III)-acremonpeptide D exhibited moderate antiviral activity against HSV-1 with EC_50_ values of 16, 8.7, and 14 μM, respectively [33] (Figure 7). Meanwhile, a new cyclic depsipeptide, acremonamide (**75**), was isolated from a marine-derived fungus *Acremonium* sp. strain CNQ-049 [34].

Six new hydroxamate siderophore cyclohexapeptides, Al (III)-acremonpeptide E (**76**), acremonpeptide E (**77**), Fe (III)-acremonpeptide E (**78**), acremonpeptide F (**79**), Al (III)-acremonpeptide F (**80**), and Fe (III)-acremonpeptide F (**81**), and one new cyclic pentapeptolide, aselacin D (**82**), together with a known compound, aselacin C, were isolated from the sponge-derived fungus *A. persicinum* F10. Compounds **76** and **80** showed pronounced antifungal activity against *Aspergillus fumigatus* and *A. niger* with a shared MIC value of 1 μg/mL, and both showed no cytotoxicity against human embryonic lung fibroblasts (MRC-5) at a concentration of 30 μM [35].

Two known cyclopeptides, (–)-ternatin and [D-Leu]-ternatin, were isolated from the EtOAc extract of the fungal strain *Acremonium* sp. SF-7394. [D-Leu]-esculetin inhibited the enzyme activity in a dose-dependent manner, with an IC_50_ value of 14.8 ± 0.3 μM [17].

### 2.3. Polyketides

A total of 60 polyketides have been reported from the genus *Acremonium* within the period, including 23 new compounds and 18 bioactive compounds.

**Figure 8:**
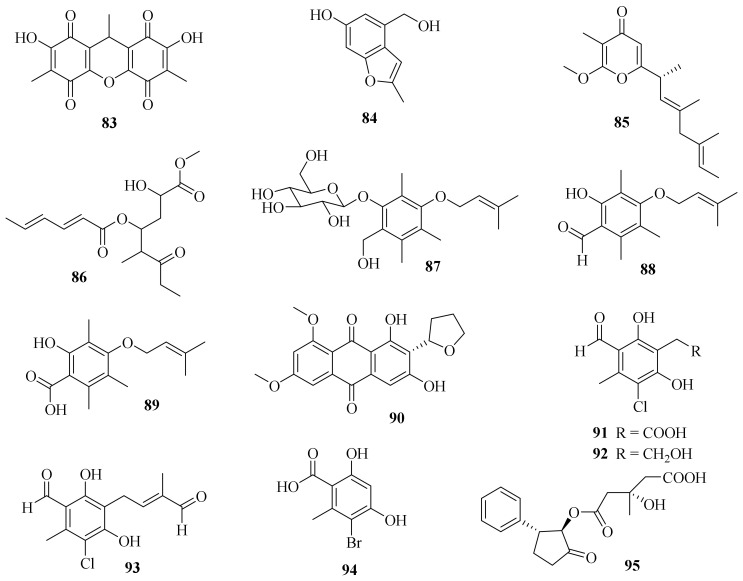
Chemical structures of polyketides (**83**–**95**).

One new dibenzoquinone, 2,7-dihydroxy-3,6,9-trimethyl-9*H*-xanthene-1,4,5,8-tetraone (**83**) (Figure 8), and a known analog, 3,3′,6,6′-tetrahydroxy-4,4′-dimethyl-1,1′-bi-*p*-benzoquinon, were obtained from the fungus *A. cavaraeanum* CA022 [36]. Meanwhile, a chemical examination of marine sponge *Mycale* sp. derived fungus *A. persicinum* KUFA 1007 led to the isolation of one new compound, acremine T (**84**) [26].

A chemical investigation on the endophytic fungus *A. citrinum* SS-g13 yielded a new γ-pyrone derivative, acrepyrone A (**85**), and three known sorbicillinoids, trichodimerol, dihydrotrichodimerol, and tetrahydrotrichodimerol [8]. A chemical investigation of the endophytic fungus *A. citrinum* MMF4 derived from the root of the mangrove plant *Kandelia obovate* resulted in the isolation of one new compound, triacremoniate (**86**), along with a known compound, acrepyrone A. Compound **86** had significant inhibitory effects on the proliferation of HeLa cells, with an IC_50_ value of 30.5 ± 1.99 μM [37].

Three new zinniol analogues, pleoniols A–C (**87**–**89**), along with a known compound were isolated from a mixed fermentation of two endophytic fungi, *Pleosporales* sp. F46 and *A. pilosum* F47, both of which originated from the pedicel of the medicinal plant *Mahonia fortune* [38]. Four dimethylated anthraquinone derivatives, including one new compound, 6,8-di-*O*-methylbipolarin (**90**), and three known compounds, aversin, 6,8-di-*O*-methylaverufin, and 6,8-di-*O*-methylnidurufin, were obtained from the marine-derived fungus *A. vitellinum* MH726097. Compound **90** showed the strongest insecticidal activity against the third instar larvae of *Helicoverpa armigera*, with a LC_50_ value of 0.72 mg/mL [39].

Three new chlorinated orsellinic aldehyde derivatives, orsaldechlorins A–C (**91**–**93**), and one new natural brominated orsellinic acid, 5-bromo-2,4-dihydroxy-6-methylbenzoic acid (**94**), along with ten known biosynthetically related derivatives were further characterized from the Beibu Gulf coral-associated fungus *A. sclerotigenum* GXIMD 02501. Most of them inhibited LPS-induced NF-κB activation in RAW 264.7 cells at a concentration of 20 μM. Notably, compounds **91** and **92** showed inhibitions of RANKL-induced osteoclast differentiation in bone marrow macrophages without cytotoxicity [40].

One new compound, fusidione (**95**), along with a known one, microperfuranone, were isolated from the sea-water-derived fungus *A. fusidioides* RZ01. Fusidione displayed inhibitory activity against HL-60 cells with an IC_50_ value of 44.9 μM [41].

**Figure 9:**
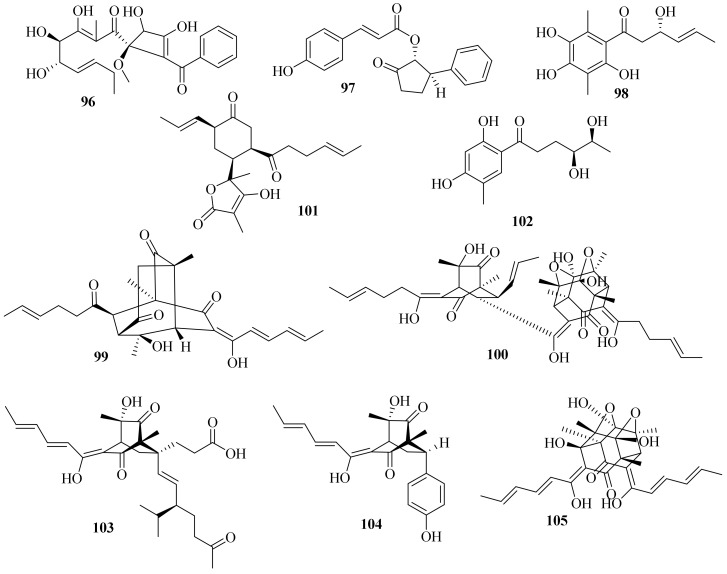
Chemical structures of polyketides (**96**–**105**).

A new benzoyl compound, 1-(2′-benzoyl-3,4-dihydroxy-1′-methoxycyclobut-2′-enyl)-3,4,5-trihydroxy-2-methylnona-2,6-dien-1-one (**96**) (Figure 9), was obtained from the endophytic fungus *Acremonium* sp. of *Garcinia griffithii* [42]. One new polyketide, acrefurcatone A (**97**), was isolated from the deep-sea cold-seep sediment-derived fungus *A. furcatum* CS-280, which showed strong activity against *Pseudomonas aeruginosa* with an MIC value of 8 μg/mL [24].

A chemical investigation of marine sediment-derived fungus *Acremonium* sp. resulted in the isolation of two new compounds, 3(*S*)-hydroxy-1-(2,4,5-trihydroxy-3,6dimethylphenyl)-hex-4*E*-en-1-one (**98**) and acremonilactone (**99**), along with eight known compounds. Among them, (2*E*,4*E*)-1-(2,6dihydroxy-3,5-dimethyl-phenyl) hexa-2,4-dien-1-one, sorbicillin, and tetrahydrotrichodimerol showed inhibitory activity against *S. aureus*, with a shared MIC value of 128 μg/mL. In addition, compounds **98** and trichodimerol showed 2,2-diphenyl-1-trinitrophenylhydrazine (DPPH) free radical scavenging activity with inhibition rates of 96.50% and 84.95% at a concentration of 0.5 mg/mL, respectively [43].

A chemical investigation of the terrestrial plant *Fructus mori* derived *A. citrinum* SS-g13 produced three new sorbicillinoids, trisorbicillinone E (**100**), acremosorbicillinoids A and B (**101** and **102**), and one new natural product, 2*S*,3*S*-acetyl-*β*-methyltryptophan, along with eight known sorbicillinoids. Among them, dihydrobisvertinolone showed significant cholesterol efflux-enhancing activity [44].

Moreover, three new sorbicillinoid derivatives, acresorbicillinols A–C (**103**–**105**), along with five known compounds were obtained from the marine-derived fungus *A. chrysogenum* C10. Compounds **104** and **105** displayed moderate activity against *S. aureus* and *Cryptococcus neoformans* with IC_50_ values of 86.93 ± 1.72 and 69.06 ± 10.50 μM, respectively. Moreover, compound **105** demonstrated strong DPPH free radical scavenging activity, with the IC_50_ value ranging from 11.53 ± 1.53 to 60.29 ± 6.28 μM in 24 h [45]. A chemical investigation of the deep-sea-derived *A. alternatum* provided two known bisorbicillinoids, tetrahydrotrichodimerol and dihydrotrichodimerol [46].

### 2.4. Steroids, Amides, or Alkaloids

#### 2.4.1. Steroids

A total of eighteen steroids have been discovered from the genus *Acremonium* during the period 2016–2023, including four new compounds as well as five bioactive compounds.

**Figure 10:**
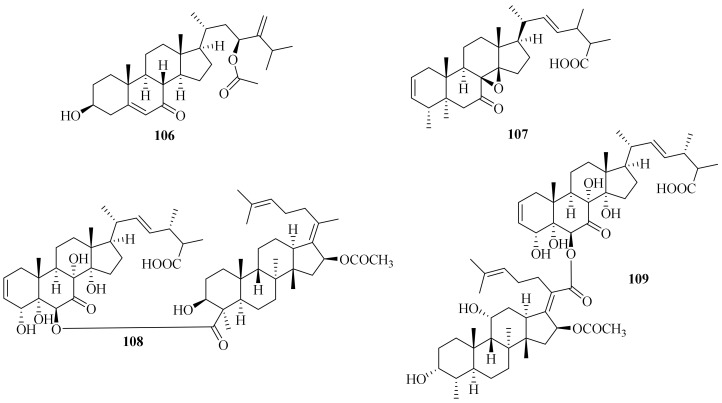
Chemical structures of steroids (**106**–**109**).

A new steroid acremocholone (**106**) (Figure 10) and three known analogs, (22*E*)5α,8α-epidioxyergosta-6,22-dien-3β-ol, (22*E*, 24*R*)3β,5α,9α,14α-tetrahydroxyergosta-7,22-dien-6-one, and (22*E*, 24*R*)-3β-hydroxy-5,9-epoxyergosta-7,22-dien6-one, were obtained from the marine mesophotic zone ciocalypta sponge-associated fungus *Acremonium* sp. NBUF150. Particularly, compound **106** showed antibacterial activity against *Vibrio scophthalmi*, *V. shilonii*, and *V. brasiliensis* with a shared MIC value of 8 μg/mL. (22*E*)5α,8α-epidioxyergosta-6,22-dien-3β-ol inhibited the growth of *V. shilonii* and *V. brasiliensis* at 8 μg/mL and 32 μg/mL, respectively. Moreover, (22*E*,24*R*)-3β-hydroxy-5,9-epoxyergosta-7,22-dien6-one inhibited the growth of *V. brasiliensis* at 16 μg/mL [47].

A new compound, (22*E*)-25-carboxy-8β,14β-epoxy-4α,5α-dihydroxyergosta-2,22-dien-7-one (**107**), along with a known compound, 5α,8α-epidioxy ergosta-6,22-diene-3β-ol, were isolated from the fermentation products of the marine-sourced fungus *A. fusidioides* RZ01. Compound **107** showed inhibitory activity against HL-60 cells with an IC_50_ value of 16.6 μM [41].

Two new heterodimers, acremonidiols B and C (**108** and **109**), and four biosynthetically related known compounds were isolated from *A. pilosum* F47 [48]. Meanwhile, four known steroids, (22*E*,24*R*)-ergosta-5,7,22-trien-3β-ol, ergosterol endoperoxide, 11-*O*-acetyl-NGA0187, and NGA0187, were obtained from *A. alternatum* [46]. Ergosterol and ergosterol 5,8-endoperoxide were isolated from the culture of sponge-associated fungus *A. persicum* KUF1007 [26].

The sterol 3β,5α,6β,7α-tetrahydroxyergosta-8(14),22-diene was isolated from the liquid culture of *A. persicum*. Its antiproliferative potential was found to be comparable to or even stronger than that of commonly used anticancer drugs in breast cancer and colon cancer cell lines T-47 D and WiDr [49].

#### 2.4.2. Amides

A total of nine amides have been discovered from the genus *Acremonium* during the period 2016–2023, including four new compounds and three bioactive compounds.

**Figure 11:**
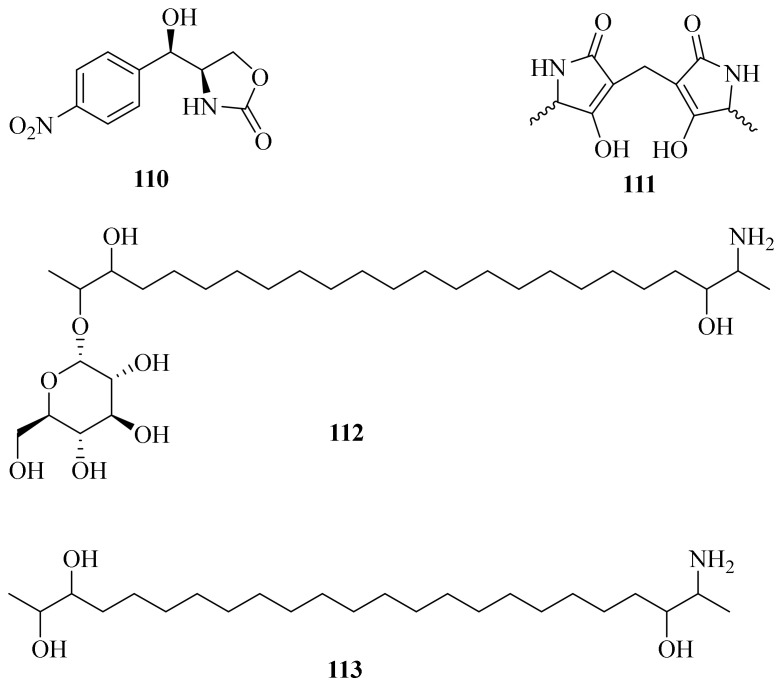
Chemical structures of amides (**110**–**113**).

Three chloramphenicol derivatives, including one new natural product, 4*R*-(1*R*-hydroxy-(4-nitrophenyl)-methyl)-1,3-oxazolidin-2-one (**110**) (Figure 11), were isolated from a marine alga-derived fungus *A. vitellinum* MH726097. Compound **110** indicated insecticidal activity against *Helicoverpa armigera* with an LC_50_ value of 0.56 ± 0.03 mg/mL, while chloramphenicol and corynecin-I exhibited weak activity with LC_50_ values of 0.93 ± 0.05 and 0.91 ± 0.06 mg/mL, respectively [50].

A new compound, dietziamide C (**111**), was obtained from the mangrove-derived fungus *A. citrinum* MMF4 [37]. Meanwhile, one new deoxysphingoid derivative, named hypoculoside (**112**), along with a new aglycone derivative, hypoculine (**113**), were isolated from the fungus *Acremonium* sp. F2434. Compound **112** completely inhibited the growth of *C. albicans* with an IC_50_ value of 7.6 μM. Hypoculoside inhibited the growth of *Saccharomyces cerevisiae* cells with an IC_50_ value of 7.2 μM and also inhibited the growth of Gram-positive bacteria *S. aureus* with an IC_50_ value of 11.7 μM. Meanwhile, hypoculoside showed cytotoxicity against human lung and pancreatic cancer cell lines (IC_50_ = 9–14 μM) [51].

A known metabolite pseurotin A was isolated from the EtOAc extract of the fungal strain *Acremonium* sp. SF7394 [17]. Moreover, two ceramides, lactariamide B and (2*S*,2′*R*,3*R*,4*E*,8*E*,3′*E*)-2-(2′-hydroxy-3′-octadecenoylamino)-9-methyl-4,8-octadecadiene-l,3-diol, were obtained from *A. alternatum* [46].

#### 2.4.3. Alkaloids

A total of nine alkaloids have been reported from the genus *Acremonium* during the period 2016–2023, including two new compounds and three bioactive compounds.

**Figure 12:**
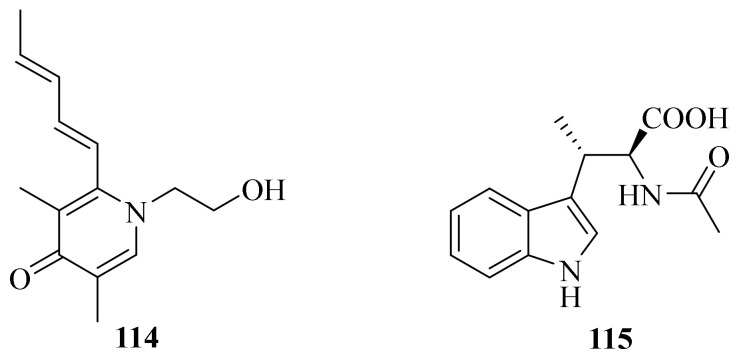
Chemical structures of alkaloids (**114** and **115**).

Two new alkaloids, acremokaloid A (**114**) and 2*S*, 3*S*-acetyl-β-methyltryptophan (**115**), were isolated from an endophytic fungus *A. citrinum* SS-g13 [44] (Figure 12). Moreover, a known compound, β-Adenosine, was obtained from the mangrove-derived fungus *A. citrinum* MMF4 [37].

Three rare 4-hydroxy-2-pyridone alkaloids, campyridones A and D, ilicicolin H, and one phenazine alkaloid, phenazine-1-carboxylic acid, were isolated from the coral-associated fungus *A. sclerotigenum* GXIMD 02501. Campyridone A and ilicicolin H showed cytotoxicity against two prostate cancer cell lines, with IC_50_ values of 17.6 ± 1.3 and 5.5 ± 1.2 μM for PC-3, and 25.4 ± 1.7 and 11.9 ± 1.3 μM for 22Rv1, respectively. In addition, phenazine-1-carboxylic acid showed anti-*Vibrio* activity, including *V. parahemolyticus*, *V. alginolyticus*, *V. owensii*, and *V. coralliilyticus*, with MIC values ranging from 0.047 to 0.067 mg/mL, and showed inhibition of LPS-induced NF-κB activation at 10 μM [52]. A chemical investigation of marine sediment-derived *Acremonium* sp. resulted in the isolation of one known compound, *N*-(2-hydroxyphenyl)-acetamide [43].

## 3. Comprehensive Overview and Conclusions

**Figure 13:**
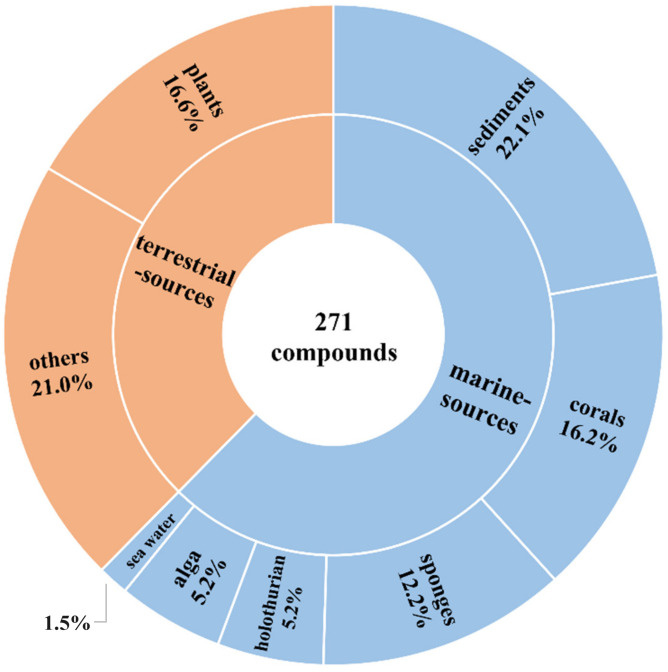
The habitat distribution of these reviewed *Acremonium* fungi from December 2016 to September 2023.

In this review, the sources, structural diversity, and biological activity of secondary metabolites from *Acremonium* fungi are summarized covering a period of time comprising the period between Dec 2016 and Sep 2023. A total of 271 compounds were obtained from the genus *Acremonium*. Among them, 115 were characterized as new compounds (42%) (Table 1). Notably, 169 compounds were predominantly marine-sourced and 77 ones were characterized as new compounds, accounting for nearly 67% of all new compounds. Most of the reviewed *Acremonium* fungi were isolated from marine habitats or terrestrial sources. Remarkably, the top three marine sources of these reviewed *Acremonium* fungi were sediments (22%), corals (16%), and sponges (12%) (Figure 13).

**Figure 14:**
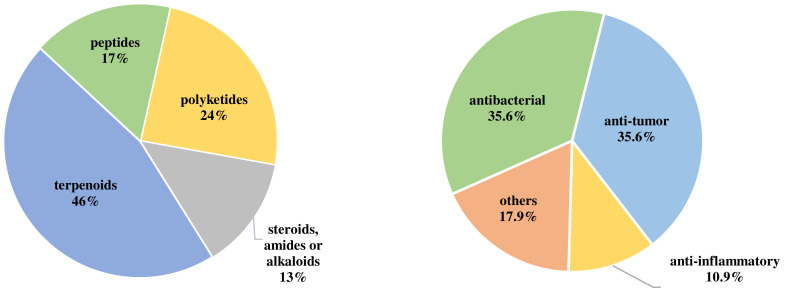
The structural diversity (**left**) and bioactivities (**right**) of secondary metabolites in *Acremonium* fungi from December 2016 to September 2023.

The chemical structures of the 115 recently reported secondary metabolites from *Acremonium* fungi can mainly be classified into four types, including terpenoids (46%), polyketides (24%), peptides (17%), and others (13%) consisting of steroids, amides, and alkaloids (Figure 14). However, among these 115 new compounds, terpenoids predominantly accounted for 42%, while polyketides, peptides, and other types accounted for 20%, 29%, and 9%, respectively. Moreover, it is worth noting that nearly 37.3% (101 compounds) showed broad-spectrum biological activities, including insecticidal, antibacterial, cytotoxic, enzyme inhibition, antiviral, anti-inflammatory, antioxidant, and antimalarial activities. Notably, antibacterial (35.6%), cytotoxic (35.6%), and anti-inflammatory (10.9%) represent the top three bioactivities.

In summary, widely distributed *Acremonium* fungi have hitherto been proven to be vital sources of novel and diverse secondary metabolites with a broad range of biological activities, revealing their great untapped potential in medicinal, agrochemical, and industrial applications. However, for most of these isolated compounds, the lack of deep pharmacological mechanisms as well as comprehensive pharmacokinetic evaluation limit their applications. Overall, this review will shed light on the further pharmacological investigation and medicinal utilization of these valuable secondary metabolites from this genus and will continuously arouse high interest in natural product chemistry, synthetic chemistry, pharmacology, and medicinal chemistry.

## Figures and Tables

**Table 1:** Recently reported new compounds from the genus *Acremonium* (December 2016 to September 2023).

Compounds	Producing Strains	Sources	Biological Activities	Ref.
acremeremophilanes A–O (**1**–**15**)	*Acremonium* sp. TVG-S004-0211	deep-sea sediments	**2**–**5**, **14**: LPS-induced NO production (IC_50_: 8–45 μM)	[15]
marinobazzanan (**16**)	*Acremonium* sp. CNQ-049	marine sediments	anti-tumor	[16]
isocordycepoloside A (**17**)	*Acremonium* sp. SF-7394	an unidentified lichen	-	[17]
4-((*Z*)-but-2-enoyloxy)-12, 13-epoxytrichotheca-7, 9-diene (**18**) 4-((*Z*)-but-2-enoyloxy)-12, 13-epoxy-16-hydroxytrichothec-9ene (**19**) 4-((*Z*)-but-2-enoyloxy)-8-chloro-12-hydroxy-7, 13epoxytrichothec-9-ene (**20**)	*A. crotocinigenum* BCC 20012	the petiole of the brackish water palm	-	[18]
virescenosides Z9–Z18 (**21**–**30**)	*A. striatisporum* KMM 4401	holothurian	-	[12]
acrepseudoterin (**31**)	*Acremonium* sp. SF-7394	an unidentified lichen	PTP1B inhibitor (IC_50_: 22.8 ± 1.1 μM)	[17]
acremochlorins A–M (**32**–**44**)	*A. sclerotigenum* GXIMD 02501	coral *Pocillopora damicornis*	**32**, **36**–**38**, **42**–**44**: Cytotoxic (MDA-MB-231 and MDA-MB-468) (IC_50_: 0.48–45 μM)	[10]
acremochlorin N (**45**) 3-phenylcyclopentane-1,2-diol (±-**46**)	*A. furcatum* CS-280	marine sediments	**45**–**46**: anti-*Vibrio*	[24]
acremine S (**47**)	*A. persicinum* KUFA 1007	marine sponge *Mycale* sp.	butyrylcholine esterase inhibiton	[26]
hexahydroacremonintriol (**48**)	*A. masseei* CICY026	plant litter	insecticidal activity (settling inhibition: 48–67%)	[27]
acremonidiol A (**49**)	*A. pilosum* F47	the pedicel of the Chinese medicinal plant *Mahonia fortunei*	-	[28]
acremopeptin (**50**)	*Acremonium* sp. PF1450	sediments	-	[29]
acremotins A–D (**51**–**54**)	*A. persicinum* SC0105	sediments	**54**: antibacterial (MIC: *S. aureus* 12.5 μg/mL, MRSA 6.25 μg/mL), anti-tumor (A549, HeLa, HepG2; IC_50_: 1.2–21.6 μM)	[30]
acremopeptaibols A–F (**55**–**60**)	*Acremonium* sp. IMB18-086	the sponge *Haliclona* sp.	**55**, **59**: antibacterial (*S. aureus*, MRSA, *B. subtilis*, *C. albicans*; MIC: 16–64 μM)	[25]
emerimicins V–X (**61**–**66**)	*A. tubakii* MT053262	sediments	**61**–**62**: toxic to zebrafish embryos. **61**: antibacterial (MIC: *E. faecalis* 64 μg/mL, MRSA 32 μg/mL, vancomycin-resistant *E. faecium* 64 μg/mL)	[31]
acrepeptins A–D (**67**–**70**)	*Acremonium* sp. NTU492	marine alga *Mastophora rosea*	**67** and **68**: LPS-induced NO production (IC_50_: 12.0 ± 2.3, 10.6 ± 4.0 mM)	[32]
acremonpeptides A–D (**71**–**74**)	*A. persicinum* SCSIO 115	marine sediments	**71** and **72**: antiviral activity (HSV-1, EC_50_: 16, 8.7 μM)	[33]
acremonamide (**75**)	*Acremonium* sp. strain CNQ-049	marine sediments	-	[34]
Al (III)-acremonpeptide E (**76**) Acremonpeptide E (**77**) Fe (III)-acremonpeptide E (**78**) acremonpeptide F (**79**) Al (III)-acremonpeptide F (**80**) Fe (III)-acremonpeptide F (**81**) aselacin D (82)	*A. persicinum* F10	marine sponge *Phakellia fusca*	antifungal **76** (*A. fumigatus* MIC: 1 μg/mL) **80** (*A. niger* MIC: 1 μg/mL)	[35]
2,7-dihydroxy-3,6,9-trimethyl-9*H*-xanthene-1,4,5,8-tetraone (**83**)	*A. cavaraeanum* CA022	fruiting bodies of *Shiraia bambusicola*	-	[36]
acremine T (**84**)	*A. persicinum* KUFA 1007	marine sponge *Mycale* sp.	-	[26]
acrepyrone A (**85**)	*A. citrinum* SS-g13	the root of the plant *Fructus mori*	-	[8]
triacremoniate (**86**)	*A. citrinum*. MMF4	the root of mangrove plant *Kandelia obovata*	**86**: anti-tumor (HeLa; IC_50_: 30.46 ± 1.99 μM)	[37]
pleoniols A–C (**87**–**89**)	*Pleosporales* sp. F46 and *A. pilosum* F47	the pedicel of the medicinal plant *Mahonia fortunei*	-	[38]
6,8-di-*O*-methylbipolarin (**90**)	*A. vitellinum* MH726097	the fresh inner tissue of an unidentified marine red alga	**90**: insecticidal (*H. armigera*; LC_50_: 0.72 mg/mL)	[39]
orsaldechlorins A–C (**91**–**93**) 5-bromo-2,4-dihydroxy-6-methylbenzoic acid (**94**)	*A. sclerotigenum* GXIMD 02501	coral *Pocillopora damicornis*	**91**–**92**: inhibit osteoclast differentiation	[40]
fusidione (**95**)	*A. fusidioides* RZ01	sea water	**95**: anti-tumor (HL-60; IC_50_: 44.9 μM)	[41]
1-(2′-benzoyl-3,4-dihydroxy- 1′-methoxycyclobut-2′-enyl)-3,4,5-trihydroxy-2-methyl-nona-2,6-dien-1-one (**96**)	*Acremonium* sp	the twigs of *Garcinia griffithii*	-	[42]
acrefurcatone A (**97**)	*A. furcatum* CS-280	marine sediments	**97**: antibacterial (*P*. *aeruginosa*; MIC: 8 μg/mL)	[24]
3(*S*)-hydroxy-1-(2,4,5-trihydroxy-3,6dimethylphenyl)-hex-4E-en-1-one (**98**) acremonilactone (**99**)	*Acremonium* sp. AN-13	marine sediments	**98**: DPPH free radical scavenging (inhibition rates: 96.50%)	[43]
trisorbicillinone E (**100**) acremosorbicillinoids A and B (**101** and **102**)	*A. citrinum* SS-g13	the root of the terrestrial plant *Fructus mori*	-	[44]
acresorbicillinols A–C (**103**–**105**)	*A. chrysogenum* C10	unknown	**104**–**105**: antibacterial (*S. aureus*, *C. neoformans*; IC_50_: 86.93 ± 1.72, 69.06 ± 10.50 μM) **105**: antioxidant activity (IC_50_:11.53 ± 1.53–60.29 ± 6.28 μM)	[45]
acremocholone (**106**)	*Acremonium* sp. NBUF150	the sponge *Ciocalypta* sp.	**106**: antibacterial (*V. scophthalmi*, *V. shilonii*, *V. brasiliensis*; MIC: 8, 8, 8 μg/mL)	[47]
(22*E*)-25-carboxy-8*β*,14*β*-epoxy-4*α*,5*α*-dihydroxyergosta-2,22-dien-7-one (**107**)	*A. fusidioides* RZ01	sea water	**107**: anti-tumor (HL-60; IC_50_: 16.6 μM)	[41]
acremonidiols B and C (**108** and **109**)	*A. pilosum* F47	the pedicel of the Chinese medicinal plant *Mahonia fortune*	-	[46]
4*R*-(1*R*-Hydroxy-(4-nitrophenyl)-methyl)-1,3-oxazolidin-2-one (**110**)	*A. vitellinum* MH726097	fresh inner tissue of an unidentified marine red alga	**110**: insecticidal activity (*H. armigera*; LC_50_: 0.56 ± 0.03 mg/mL)	[50]
dietziamide C (**111**)	*A. citrinum*. MMF4	the root of mangrove plant *Kandelia obovata*	-	[37]
hypoculoside (**112**) hypoculine (**113**)	*Acremonium* sp. F2434	sediments	**112**: antibacterial (*C. albicans*, *S. aureus*, *S. cerevisiae*; IC_50_: 11.7, 7.6, 7.2 μM) **113**: anti-tumor (LUNG, PAAD; IC_50_: 9–14 μM)	[51]
acremokaloid A (**114**) 2*S*,3*S*-acetyl-*β*-methyltryptophan (**115**)	*A. citrinum* SS-g13	terrestrial plant *Fructus mori*	-	[44]

## Supplementary Materials

The following supporting information can be downloaded at https://www.mdpi.com/article/10.3390/jof10010037/s1, Table S1: Recently reported known compounds from the genus *Acremonium*, along with their structures.
