# Supplementary material for: Recent Advances in Chemistry and Bioactivities of Secondary Metabolites from the Genus Acremonium
Source: J Fungi (Basel). 2024 Jan 3;10(1):37. doi: 10.3390/jof10010037 (PMC10820033; doi:10.3390/jof10010037)
Supplement: Supplementary file 1 [file jof-10-00037-s001.zip › jof-2769783-supplementary.pdf]

Supplementary materials

Recent advances in chemistry and bioactivities of secondary metabolites from the genus *Acremonium*

Yuning Qin †, Humu Lu †, Xin Qi, Miaoping Lin, Chenghai Gao, Yonghong Liu* and Xiaowei Luo*

Institute of Marine Drugs, Guangxi Key Laboratory of Marine Drugs, Guangxi University of Chinese Medicine, Nanning, 530200, China;
* Correspondence: yonghongliu@scsio.ac.cn (Y.L.); luoxiaowei1991@126.com (X.L.)
† These authors contributed equally to this work.

Table S1 Recent reported known compounds from the genus *Acremonium* (December 2016 to September 2023).

Compounds	Producing strains	Sources	Ref.
trichodimerol, dihydrotrichodimerol, tetrahydrotrichodimerol	*Acremonium citrinum* SS-g13	the root of the plant *Fructus mori*	[8]
cylindrol B, LL-*Z*1272*ε*, ilicicolin C, ascochlorin, 4',5'-dihydro-4'-hydroxyascochlorin7, 10'-deoxy-10'*α*-hydroxyascochlorin, ascofuranone, ascofuranol, 10'-hydroxyilicicolinic acid D, cylindrocarpol, chlorocylindrocarpol, LL-*Z*1272*α*	*Acremonium sclerotigenum* GXIMD 02501	coral *Pocillopora damicornis*	[10]
guignarderemophilane B, guignarderemophilane E, eremophil-1(10),11(12)-dien-2*β*,8*β*-diol, 2-oxo-3-hydroxyeremophila-1(10),3,7(11),8-tetraen-8,12-olide, the C-2 epimer of PF1092B, the C-3 epimer of PF1092C, the C-2 epimer of PF1092C	*Acremonium* sp. TVG-S004-0211	deep-sea sediments	[15]
(−)-ternatin, [D-Leu]-ternatin, pseurotin A	*Acremonium* sp. SF-7394	an unidentified lichen	[17]

crotocin，trichothecin，8-deoxytrichothecinol B，4-((*Z*)-but-2-enoyloxy)-8,12-dihydroxy-7，13-epoxytrichothec9-ene	*Acremonium crotocinigenum* BCC 20012	the petiole of the brackish water palm	[18]
virescenoside F，virescenoside G，lactone of virescenoside G，aglycon of virescenoside A	*Acremonium striatisporum* KMM 4401	holothurian	[12]
ascofuranone，ascochlorin	*Acremonium egyptiacum*	-	[19]
ascofuranone，Rac-AF，CCB，(2*E*,6*E*)-8-(3-chloro-5-formyl-2,6-dihydroxy-4-methylphenyl)-2,6-dimethylocta-2,6-dien-1-yl pivalate，3-chloro-5-((2*E*,6*E*)-3,7-dimethyl-8-((tetrahydrofuran-2-yl)oxy)octa-2,6-dien-1-yl)-4,6-dihydroxy-2-methylbenzaldehyde，3-chloro-4,6-dihydroxy-5-((2*E*,6*E*)-8-hydroxy-3,7-dimethylnona-2,6-dien-1-yl)-2-methylbenzaldehyde，(3*E*,7*E*)-9-(3-chloro-5-formyl-2,6-dihydroxy-4-methylphenyl)-3,7-dimethylnona-3,7-dien-2-yl acetate，3-chloro-4,6-dihydroxy-2-methyl-5-((2*E*,6*E*)-3,7,11-trimethyldodeca-2,6,10-trien-1-yl)benzaldehyde，methyl(2*E*,6*E*)-8-(3-chloro-5-formyl-2,6-dihydroxy-4-methylphenyl)-2,6-dimethylocta-2,6-dienoate，ascochlorin，(2*E*,6*E*)-8-(3-chloro-5-formyl-2,6-dihydroxy-4-methylphenyl)-3,6-dimethylocta-2,6-dien-1-yl pivalate，(2*E*,6*E*)-8-(3-chloro-5-formyl-2,6-dihydroxy-4-methylphenyl)-3,6-dimethylocta-2,6-dien-1-yl isobutyrate，(2*E*,6*E*)-8-(3-chloro-5-formyl-2,6-dihydroxy-4-methylphenyl)-2-methylocta-2,6-dien-1-yl pivalate，8-(3-chloro-5-formyl-2,6-dihydroxy-4-methylphenyl)-2,6-dimethyloctyl pivalate，(*E*)-7-(3-chloro-5-formyl-2,6-dihydroxy-4-	*Acremonium sclerotigenum*	-	[20]

methylphenyl)-5-methylhept-5-en-1-yl pivalate，<br>7-(3-chloro-5-formyl-2,6-dihydroxy-4-methylcyclohexa-1,3-dien-1-yl)-5-methylheptyl pivalate，<br>(*E*)-3-chloro-4,6-dihydroxy-2-methyl-5-(3-methyloct-2-en-1-yl)benzaldehyde，<br>3-chloro-4,6-dihydroxy-2-methyl-5-(3-methyloctyl)benzaldehyde，<br>5-((6*E*)-8-(3-acetyl-5-chloro-2,6-dihydroxy-4-methylphenyl)-6-methylocta-2,6-dien-2-yl)-2,2-dimethyldihydrofuran-3(2*H*)-one，<br>(2*E*,6*E*)-8-(3-chloro-2,6-dihydroxy-5-(hydroxymethyl)-4-methylphenyl)-2,6-dimethylocta-2,6-dien-1-yl pivalate，<br>4-chloro-5-((2*E*,6*E*)-7-((*R*)-5,5-dimethyl-4-oxotetrahydrofuran-2-yl)-3-methylocta-2,6-dien-1-yl)-6-hydroxy-4-methoxy-2-methylbenzaldehyde，<br>colletorin B，<br>(*E*)-3-chloro-5-(3,7-dimethylocta-2,6-dien-1-yl)-4,6-dihydroxy-2-methylbenzaldehyde			
3-bromoascochlorin	*Acremonium sclerotigenum* GXIMD 02501	coral *Pocillopora damicornis*	[21]
ascofuranone	*Acremonium egyptiacum*	-	[23]
ascochlorin，<br>cylindrol B，<br>ilicicolin E，<br>ilicicolin C，<br>LL-*Z*1272*ε*，<br>10′-deoxy-10′*α*-hydroxyascochlorin，<br>ascofuranol，<br>ascofuranone，<br>chlorocylindrocarpol	*Acremonium furcatum* CS-280	sediment samples	[24]
ascochlorin，<br>10′-deoxy10′*α*-hydroxyascochlorin，<br>4′,5′-dihydro-4′-hydroxyascochlorin，<br>ascofuranone，<br>PF1171A	*Acremonium* sp. IMB18-086	the sponge *Haliclona* sp.	[25]
lumichrome，<br>ergosterol，	*Acremonium persicinum*	marine sponge	[26]

ergosterol 5,8-endoperoxide	KUFA 1007	*Mycale* sp.	
acremonin A glucoside	*Acremonium masseei* CICY026	plant litter	[27]
fusidic acid，<br>16-desacetylfusidic acid，<br>3β,20-dihydroxy-protosta-16,24-dien-29-oic acid	*Acremonium pilosum* F47	the pedicel of the Chinese medicinal plant *Mahonia fortunei*	[28]
adenopeptin	*Acremonium* sp. PF1450	sediments	[29]
XR586	*Acremonium persicinum* SC0105	sediments	[30]
destruxin B，<br>guangomide A，<br>guangomide B	*Acremonium* sp. NTU492	marine alga *Mastophora rosea*	[32]
Al(III)−acremonpeptide D	*Acremonium persicinum* SCSIO 115	marine sediments	[33]
Ga (III) - Acremonpeptide E，<br>Ga (III) - Acremonpeptide F，<br>aselacin C	*Acremonium persicinum* F10	marine sponge *Phakellia fusca*	[35]
3,3',6,6'-tetrahydroxy-4,4'-dimethyl-1,1'-bi-p-benzoquinon	*Acremonium cavaraeanum* CA022	fruiting bodies of *Shiraia bambusicola*	[36]
β-adenosine，<br>acrepyrone A	*Acremonium citrinum.* MMF4	the root of mangrove plant *Kandelia obovata*	[37]
(3*R*,4*R*)-3,4-dihydro-3,4,8-trihydroxy-1(2*H*)-naphthalenone	*Pleosporales* sp. F46 and *Acremonium pilosum* F47	the pedicel of the medicinal plant *Mahonia fortunei*	[38]
aversin，<br>6,8-di-*O*-methylaverufin，	*Acremonium vitellinum*	the fresh inner tissue	[39]

6,8-di-*O*-methylnidurufin	MH726097	of an unidentified marine red alga	
orsellinic acid，<br>ethyl orsellinate，<br>5-chloroorsellinic acid，<br>orcinol，<br>*O*-methylorcinol，<br>2-chloro-3,5-dihydroxytoluene，<br>aryl bromide，<br>ethyl 4-hydroxyphenylacetate，<br>(3*S*)-3,5-dimethyl-8-methoxy-3,4-dihydro-1H-isochromen-6-ol，<br>nectriatone C	*Acremonium sclerotigenum* GXIMD 02501	coral *Pocillopora damicornis*	[40]
5*α*,8*α*-epidioxy ergosta-6,22-diene-3*β*-ol，<br>microperfuranone	*Acremonium fusidioides* RZ01	sea water	[41]
sorbiquinol，<br>(2*E*,4*E*)-1-(2,6dihydroxy-3,5-dimethyl-phenyl)hexa-2,4-dien-1-one，<br>clavatol，<br>sorbicillin，<br>4-hydroxybenzoic acid,<br>*N*-(2-hydroxyphenyl)-acetamide，<br>trichodimerol，<br>dihydrotrichodimerol，<br>tetrahydrotrichodimerol	*Acremonium* sp. AN-13	marine sediments	[43]
trichotetronine，<br>dihydrotrichotetronine，<br>10,11-dihydrobislongiquinolide，<br>10,11,16,17-tetrahydrobislongiquinolide，<br>bisvertinolone，<br>dihydrobisvertinolone，<br>tetrahydrobisvertinolone，<br>penicillone B	*Acremonium citrinum* SS-g13	the root of the terrestrial plant *Fructus mori*	[44]
trichotetronine，<br>trichodimerol，<br>demethyltrichodimerol，<br>trichopyrone，<br>oxosorbicillinol	*Acremonium chrysogenum* C10	-	[45]
tetrahydrotrichodimerol，<br>dihydrotrichodimerol，<br>lactariamide B，	*Acremonium alternatum*	the deep-sea sediments	[46]

(2*S*,2′*R*,3*R*,4*E*,8*E*,3′*E*)-2-(2′-hydroxy-3-octadecenoylamino)-9-methyl-4,8-octadecadiene-l,3-diol，<br>(22*E*,24*R*)-ergosta-5,7,22-trien-3*β*-ol，<br>ergosterol endoperoxide，<br>6-*O*-acetyl-NGA0187，<br>7-NGA0187			
(22*E*)5*α*,8*α*-epidioxyergosta-6,22-dien-3*β*-ol，<br>(22*E*,24*R*)3*β*,5*α*,9*α*,14*α*-tetrahydroxyergosta-7,22-dien-6-one，<br>(22*E*,24*R*)-3*β*-hydroxy-5,9-epoxyergosta-7,22-dien6-one	*Acremonium* sp. NBUF150	the sponge *Ciocalypta* sp.	[47]
fusidic acid，<br>(22*E*,24*R*)-5*α*,8*α*-epidioxy-ergosta-6,22dien-3*β*-ol，<br>(3*β*,5*α*,22*E*,24*R*)-dihydroxy-ergosta7,22-dien-6-one，<br>(3*β*,5*α*,6*β*,22*E*,24*R*)-trihydroxy-ergosta-7,22-dien-6-one	*Acremonium pilosum* F47	the pedicel of the Chinese medicinal plant *Mahonia fortune*	[48]
3*β*,5*α*,6*β*,7*α*-tetrahydroxyergosta-8(14),22-diene	*Acremonium persicinum*	fungi in plant materials, sediments and water samples	[49]
chloramphenicol，<br>corynecin-I	*Acremonium vitellinum* MH726097	fresh inner tissue of an unidentified marine red alga	[50]
campyridone D，<br>campyridone A，<br>ilicicolin H，<br>phenazine-1-carboxylic acid	*Acremonium sclerotigenum* GXIMD 02501	coral *Pocillopora damicornis*	[52]
acremine P，<br>acremine A，<br>acremine Q	*Acremonium persicinum*	the marine sponge *Anomoianthella rubra*	-

Chemical structures of recent reported known compounds from the genus *Acremonium* (December 2016 to September 2023).

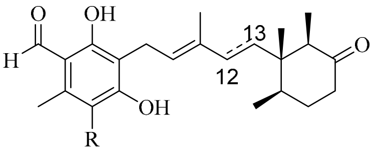

cylindrol B, R = H, $\Delta^{12(13)}$
LL-Z1272, R = H,
ilicicolin C, R = Cl,
ascochlorin, R = H, $\Delta^{12(13)}$

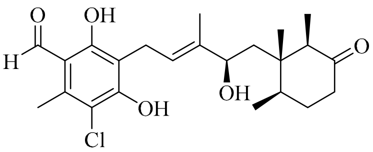

4',5'-dihydro-4'-hydroxyascochlorin7

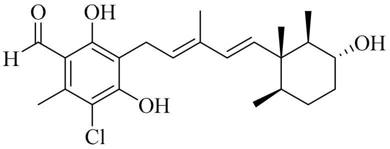

10'-deoxy-10'α-hydroxyascochlorin

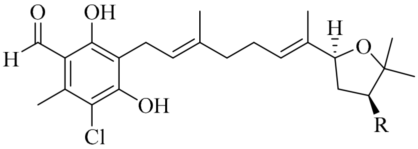

ascofuranone, R = =O
ascofuranol, R = OH

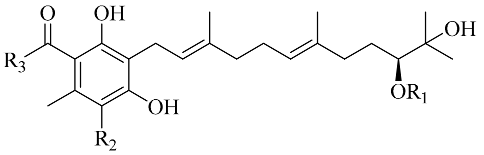

10'-hydroxyilicicolinic acid D, R_1 = H, R_2 = Cl, R_3 = OH
cylindrocarpol, $R_1 = R_2 = R_3$ = H
chlorocylindrocarpol, $R_1 = R_3$ = H, R_2 = Cl

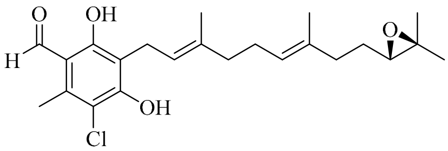

LL-Z1272α

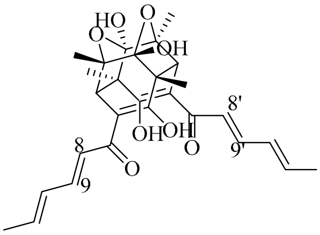

trichodimerol
dihydrotrichodimerol : 8,9-dihydro
tetrahydrotrichodimerol : 4 8,9-dihydro 8',9'-dihydro

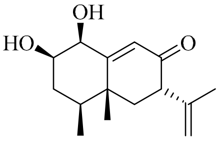

guignarderemophilane B

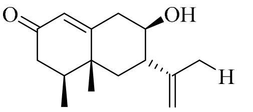

guignarderemophilane E

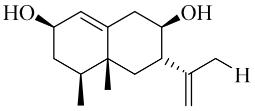

eremophil-1(10),11(12)-dien-2β,8β-diol

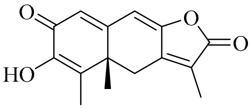

2-oxo-3-hydroxyeremophila-1(10),3,7(11),8-tetraen-8,12-olide

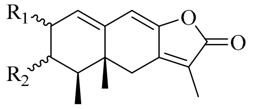

the C-2 epimer of PF1092B, R_1 = 2α-OH, R_2 = 3β-OAc
the C-3 epimer of PF1092C, R_1 = 2β-OH, R_2 = 3α-OH
the C-2 epimer of PF1092C, R_1 = 2α-OH, R_2 = 3β-OH

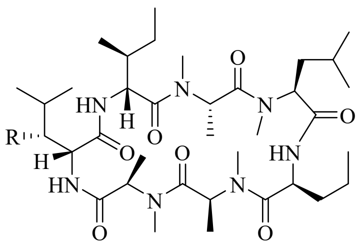

(-)-ternatin, R = OH
[D-Leu]-ternatin, R = H

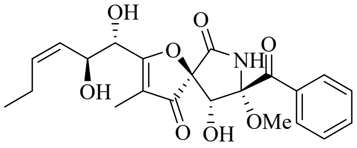

pseurotin A

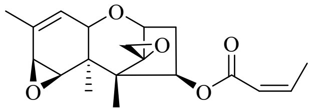

crotocin

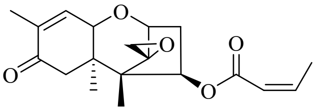

trichothecin

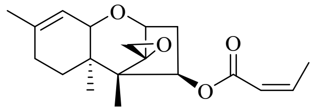

8-deoxytrichothecinol B

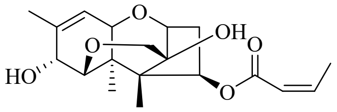

4-((*Z*)-but-2-enoyloxy)-8,12-dihydroxy-7,13-epoxytrichothec9-ene

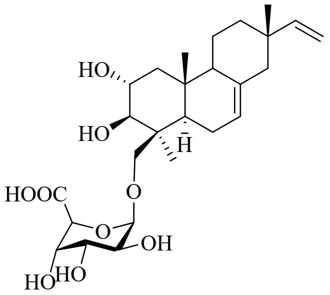

virescenoside F

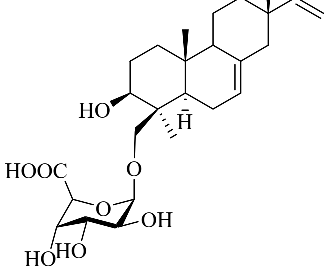

virescenoside G

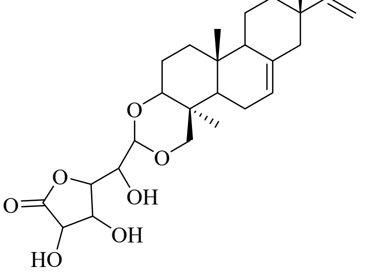

lactone of virescenoside G

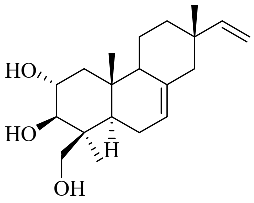

aglycon of virescenoside A

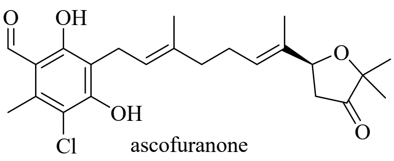

ascofuranone

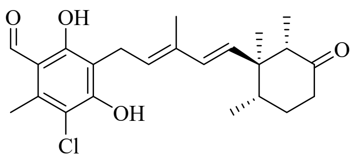

ascochlorin

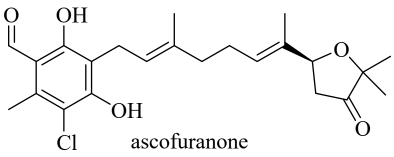

ascofuranone

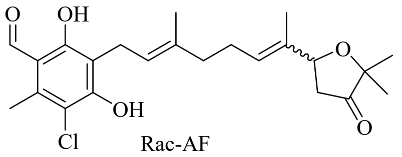

Rac-AF

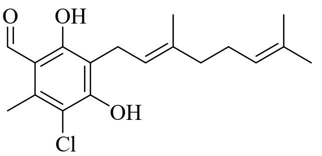

CCB

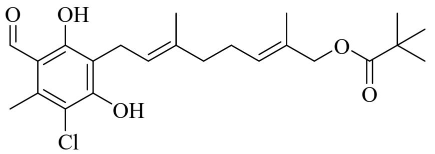

(2*E*,6*E*)-8-(3-chloro-5-formyl-2,6-dihydroxy-4-methylphenyl)-2,6-dimethylocta-2,6-dien-1-yl pivalate

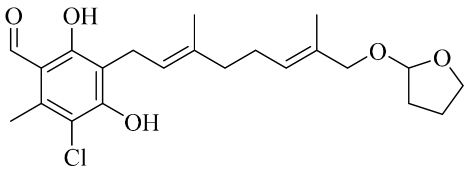

3-chloro-5-((2*E*,6*E*)-3,7-dimethyl-8-((tetrahydrofuran-2-yl)oxy)octa-2,6-dien-1-yl)-4,6-dihydroxy-2-methylbenzaldehyde

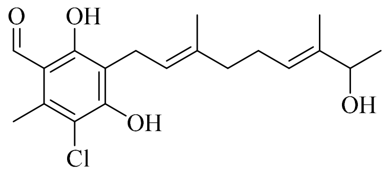

3-chloro-4,6-dihydroxy-5-((2*E*,6*E*)-8-hydroxy-3,7-dimethylnona-2,6-dien-1-yl)-2-methylbenzaldehyde

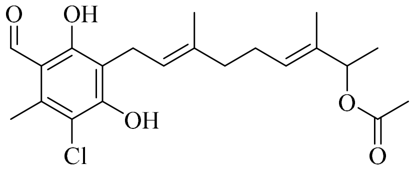

(3*E*,7*E*)-9-(3-chloro-5-formyl-2,6-dihydroxy-4-methylphenyl)-3,7-dimethylnona-3,7-dien-2-yl acetate

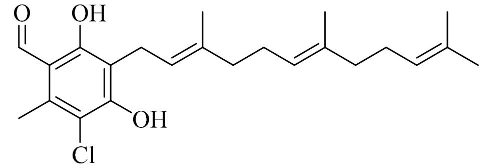

3-chloro-4,6-dihydroxy-2-methyl-5-((2*E*,6*E*)-3,7,11-trimethyldodeca-2,6,10-trien-1-yl)benzaldehyde

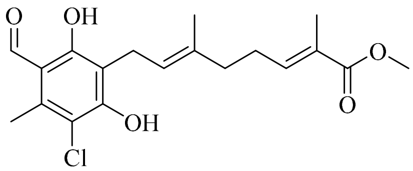

methyl (2*E*,6*E*)-8-(3-chloro-5-formyl-2,6-dihydroxy-4-methylphenyl)-2,6-dimethylocta-2,6-dienoate

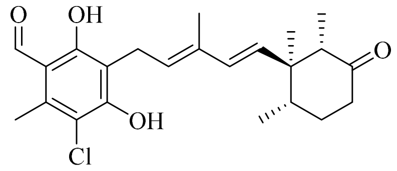

ascochlorin

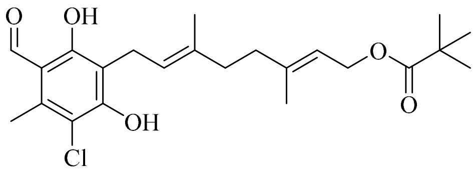

(2*E*,6*E*)-8-(3-chloro-5-formyl-2,6-dihydroxy-4-methylphenyl)-3,6-dimethylocta-2,6-dien-1-yl pivalate

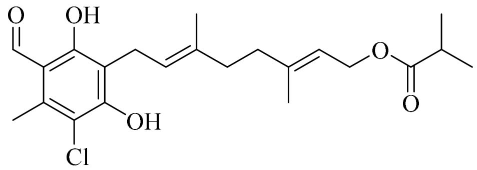

(2*E*,6*E*)-8-(3-chloro-5-formyl-2,6-dihydroxy-4-methylphenyl)-3,6-dimethylocta-2,6-dien-1-yl isobutyrate

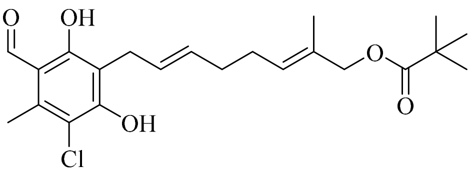

(2*E*,6*E*)-8-(3-chloro-5-formyl-2,6-dihydroxy-4-methylphenyl)-2-methylocta-2,6-dien-1-yl pivalate

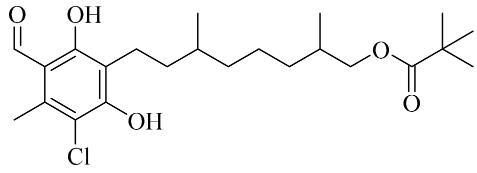

8-(3-chloro-5-formyl-2,6-dihydroxy-4-methylphenyl)-2,6-dimethyloctyl pivalate

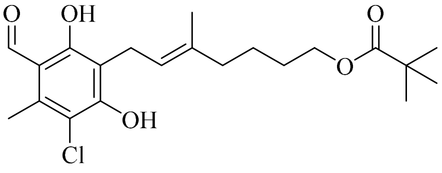

(*E*)-7-(3-chloro-5-formyl-2,6-dihydroxy-4-methylphenyl)-5-methylhept-5-en-1-yl pivalate

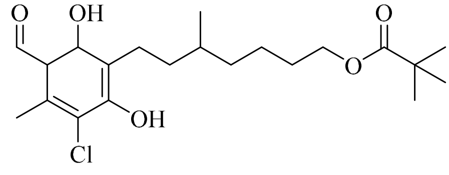

7-(3-chloro-5-formyl-2,6-dihydroxy-4-methylcyclohexa-1,3-dien-1-yl)-5-methylheptyl pivalate

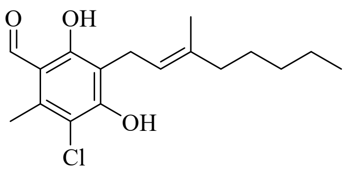

(*E*)-3-chloro-4,6-dihydroxy-2-methyl-5-(3-methyloct-2-en-1-yl)benzaldehyde

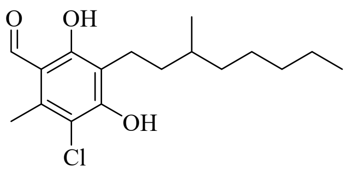

3-chloro-4,6-dihydroxy-2-methyl-5-(3-methyloctyl)benzaldehyde

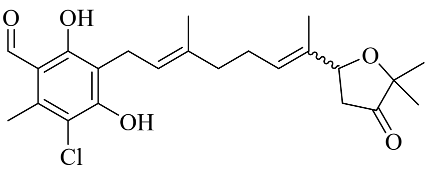

3-chloro-5-((2*E*)-7-(5,5-dimethyl-4-oxotetrahydrofuran-2-yl)-3-methylocta-2,6-dien-1-yl)-4,6-dihydroxy-2-methylbenzaldehyde

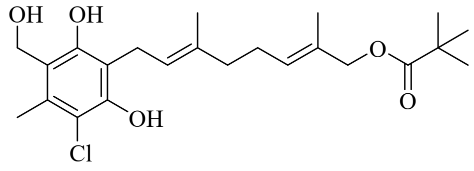

(2*E*,6*E*)-8-(3-chloro-2,6-dihydroxy-5-(hydroxymethyl)-4-methylphenyl)-2,6-dimethylocta-2,6-dien-1-yl pivalate

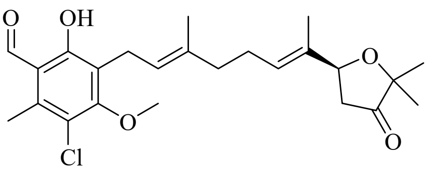

3-chloro-5-((2*E*,6*E*)-7-((*S*)-5,5-dimethyl-4-oxotetrahydrofuran-2-yl)-3-methylocta-2,6-dien-1-yl)-6-hydroxy-4-methoxy-2-methylbenzaldehyde

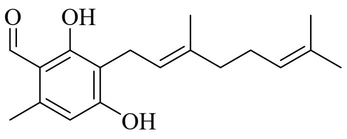

colletorin B

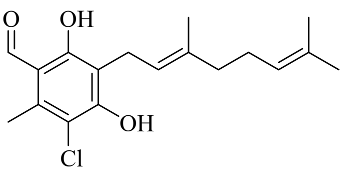

(*E*)-3-chloro-5-(3,7-dimethylocta-2,6-dien-1-yl)-4,6-dihydroxy-2-methylbenzaldehyde

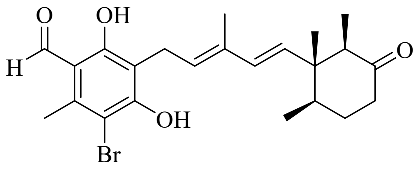

3-bromoascochlorin

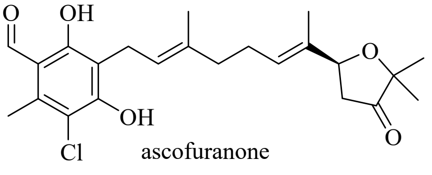

ascofuranone

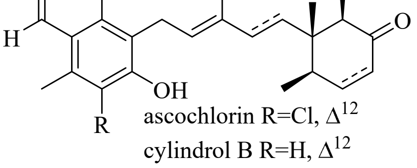

ascochlorin R=Cl, Δ^{12}
cylindrol B R=H, Δ^{12}
ilicicolin E R=Cl, $\Delta^{12, 16}$
ilicicolin C R=Cl
LL-*Z*1272ε R=H

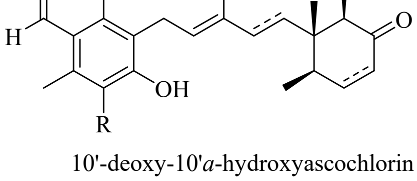

10'-deoxy-10'*a*-hydroxyascochlorin

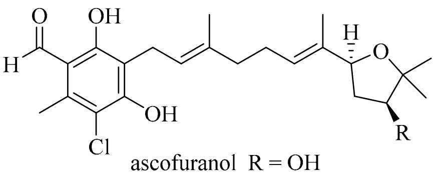

ascofuranol R = OH
ascofuranone R = =O

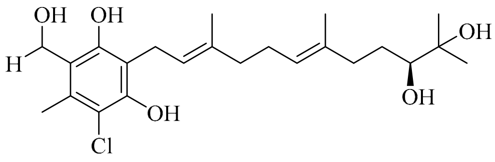

chlorocylindrocarpol

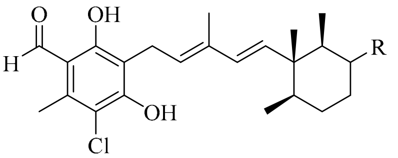

ascochlorin, R ==O
10'-deoxy10'α-hydroxyascochlorin, R = ''''OH

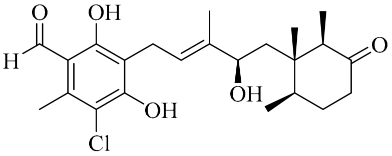

4',5'-dihydro-4'-hydroxyascochlorin

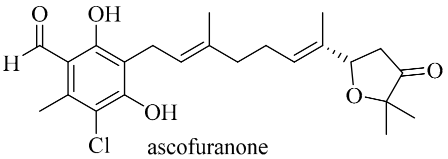

ascofuranone

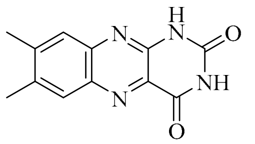

lumichrome

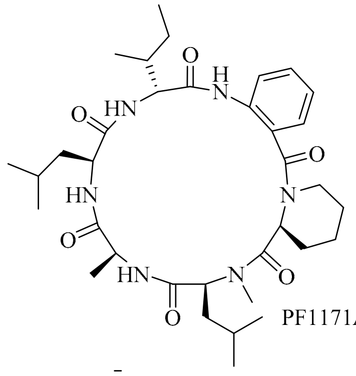

PF1171A

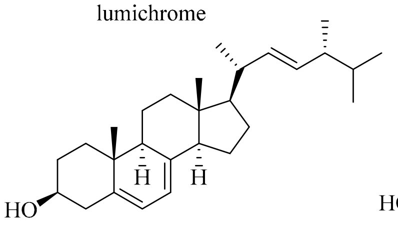

ergosterol

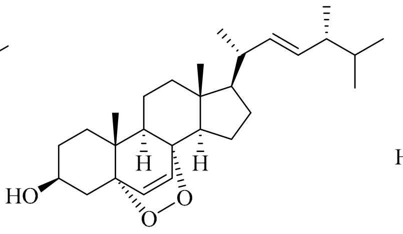

ergosterol 5,8-endoperoxide

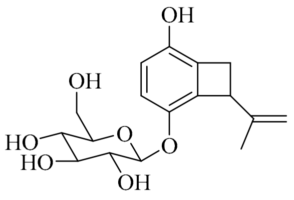

acremonin A glucoside

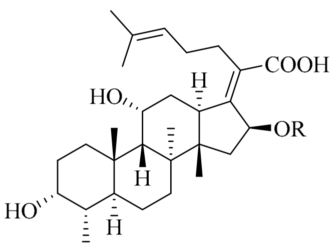

fusidic acid, R = Ac
16-desacetylfusidic acid, R = H

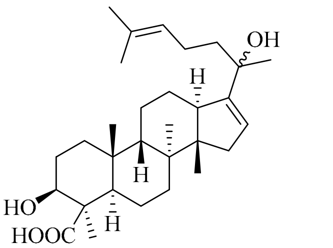

3β,20-dihydroxy-protosta-16,24-dien-29-oic acid

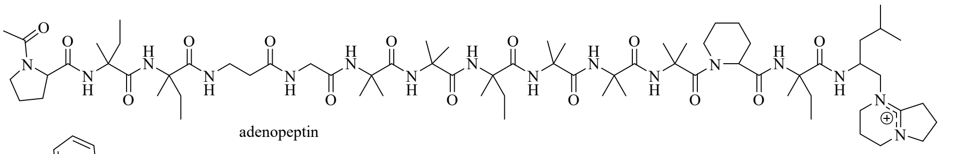

adenopeptin

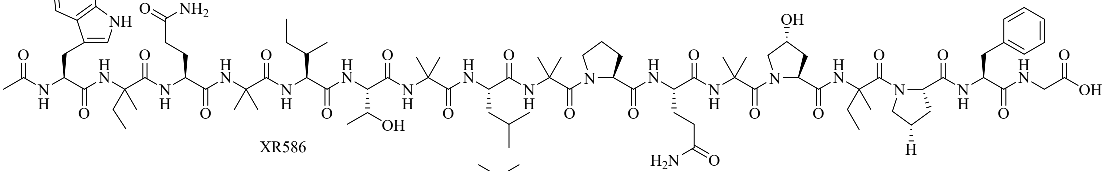

XR586

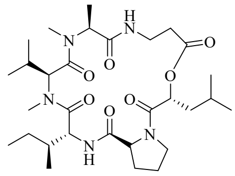

destruxin B

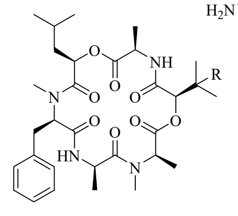

guangomide A, R = OH
guangomide B, R = H

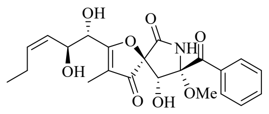

pseurotin A

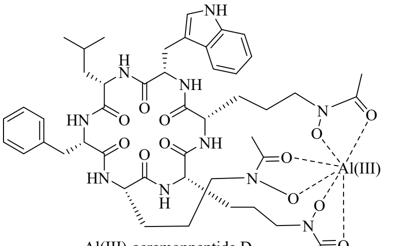

Al(III)-acremonpeptide D

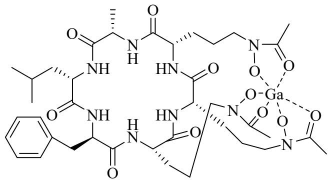

Ga (III) - Acremonpeptide E

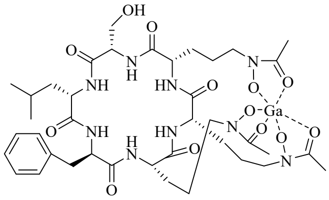

Ga (III) - Acremonpeptide F

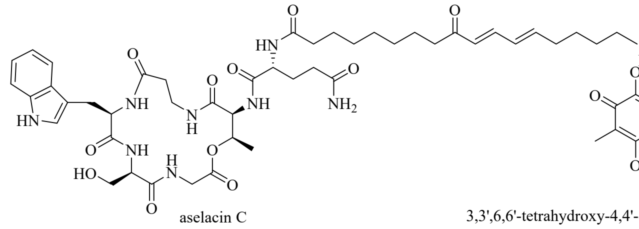

aselacin C

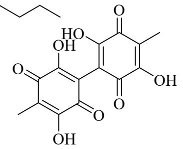

3,3',6,6'-tetrahydroxy-4,4'-dimethyl-1,1'-bi-p-benzoquinon

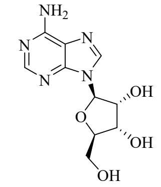

β-adenosine

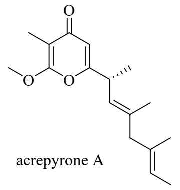

acrepyrone A

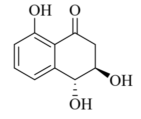

(3*R*,4*R*)-3,4-dihydro-3,4,8-trihydroxy-1(2*H*)-naphthalenone

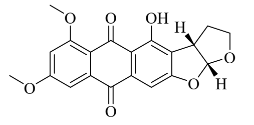

aversin

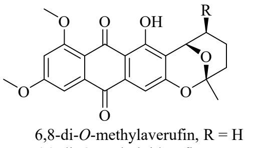

6,8-di-*O*-methylaverufin, R = H
6,8-di-*O*-methylnidurufin, R = OH

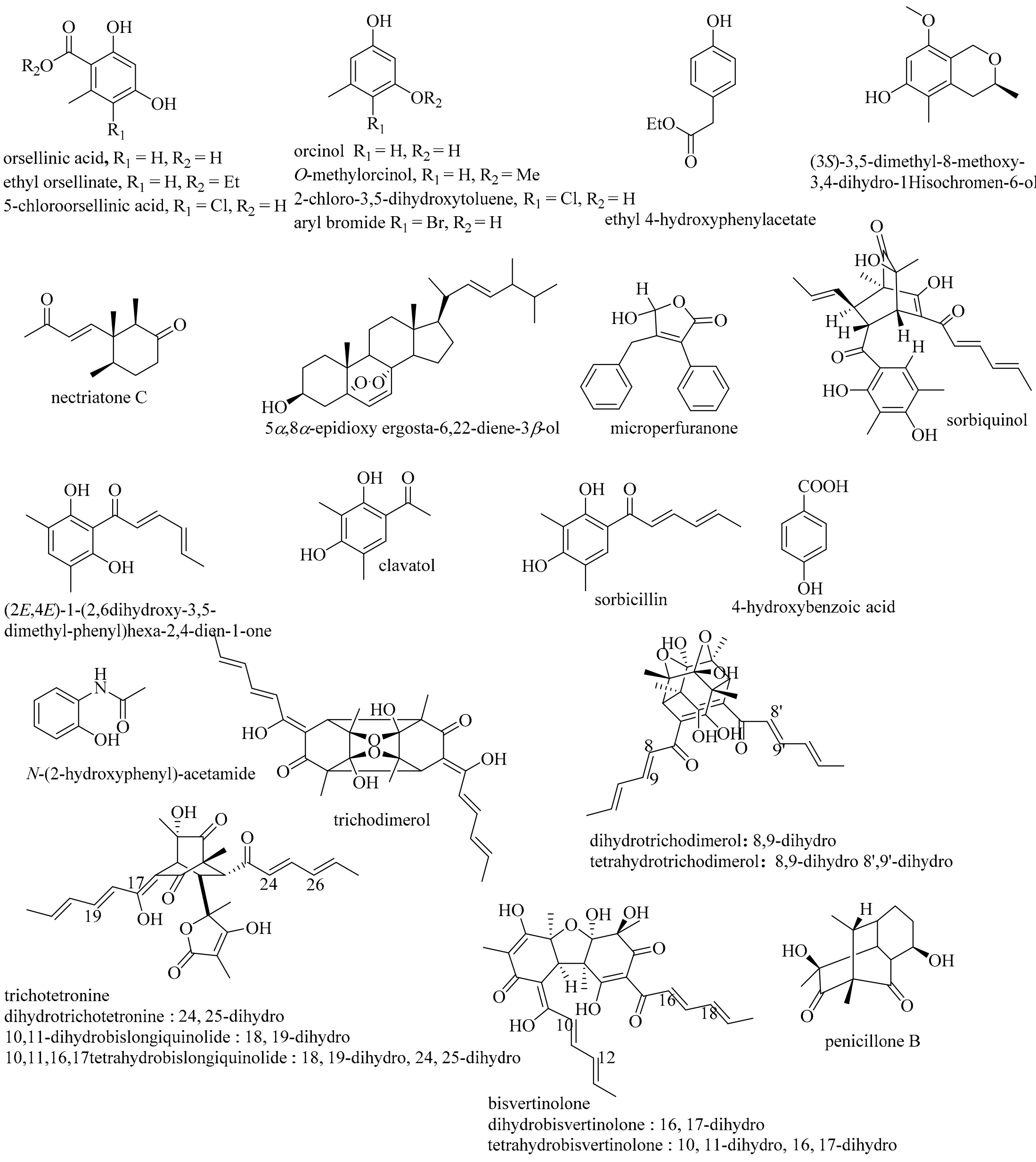
orsellinic acid, R_1 = H, R_2 = H
ethyl orsellinate, R_1 = H, R_2 = Et
5-chloroorsellinic acid, R_1 = Cl, R_2 = H
orcinol R_1 = H, R_2 = H
O-methylorcinol, R_1 = H, R_2 = Me
2-chloro-3,5-dihydroxytoluene, R_1 = Cl, R_2 = H
aryl bromide R_1 = Br, R_2 = H
ethyl 4-hydroxyphenylacetate
(3*S*)-3,5-dimethyl-8-methoxy-3,4-dihydro-1Hisochromen-6-ol
nectriatone C
5α,8α-epidioxy ergosta-6,22-diene-3β-ol
microperfuranone
sorbiquinol
(2*E*,4*E*)-1-(2,6dihydroxy-3,5-dimethyl-phenyl)hexa-2,4-dien-1-one
clavatol
sorbicillin
4-hydroxybenzoic acid
N-(2-hydroxyphenyl)-acetamide
trichodimerol
dihydrotrichodimerol: 8,9-dihydro
tetrahydrotrichodimerol: 8,9-dihydro 8',9'-dihydro
trichotetronine
dihydrotrichotetronine : 24, 25-dihydro
10,11-dihydrobislongiquinolide : 18, 19-dihydro
10,11,16,17tetrahydrobislongiquinolide : 18, 19-dihydro, 24, 25-dihydro
bisvertinolone
dihydrobisvertinolone : 16, 17-dihydro
tetrahydrobisvertinolone : 10, 11-dihydro, 16, 17-dihydro
penicillone B

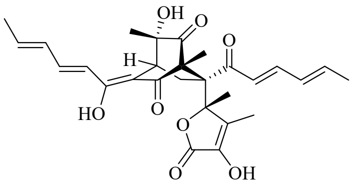

trichotetronine

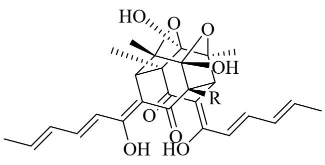

trichodimerol, R = CH_3
demethyltrichodimerol, R = H

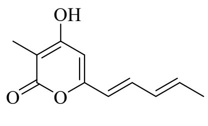

trichopyrone

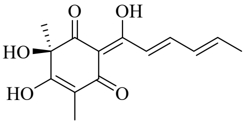

oxosorbicillinol

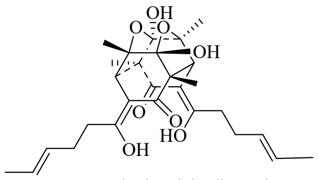

tetrahydrotrichodimerol

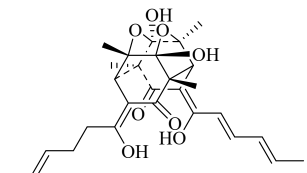

dihydrotrichodimerol

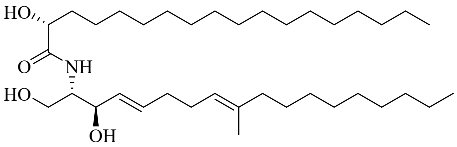

lactariamide B

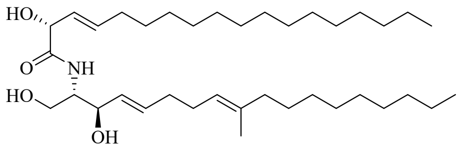

(2*S*,2'*R*,3*R*,4*E*,8*E*,3'*E*)-2-(2'-hydroxy-3'-octadecenoylamino)-9-methyl-4,8-octadecadiene-l,3-diol

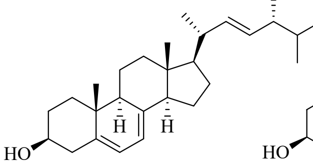

(22*E*,24*R*)-ergosta-5,7,22-trien-3β-ol

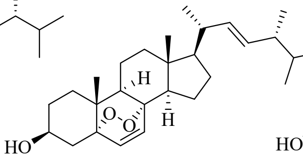

ergosterol endoperoxide

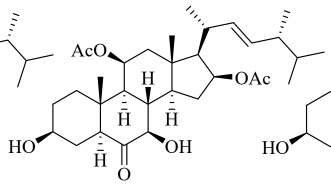

11-*O*-acetyl-NGA0187

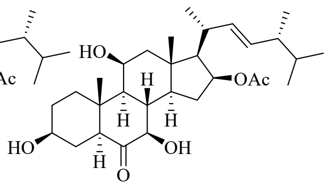

NGA0187

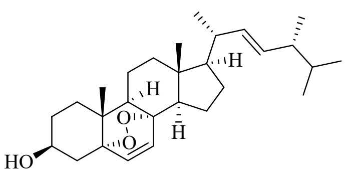

(22*E*)5α,8α-epidioxyergosta-6,22-dien-3β-ol

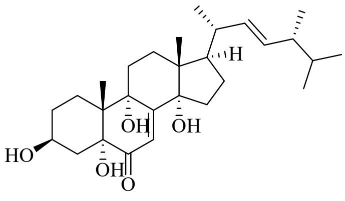

(22*E*,24*R*)3β,5α,9α,14α-tetrahydroxyergosta-7,22-dien-6-one

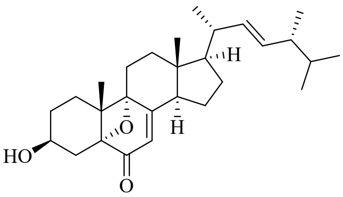

(22*E*,24*R*)-3β-hydroxy-5,9-epoxyergosta-7,22-dien6-one

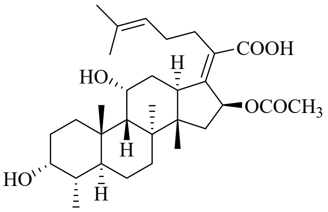

fusidic acid

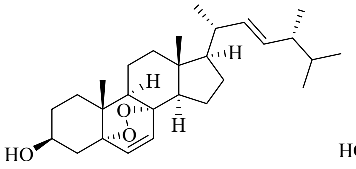

(22*E*,24*R*)-5α,8α-epidioxy-ergosta-6,22dien-3β-ol

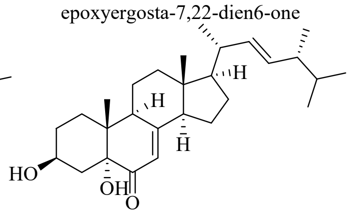

(3β,5α,22*E*,24*R*)-dihydroxy-ergosta7,22-dien-6-one

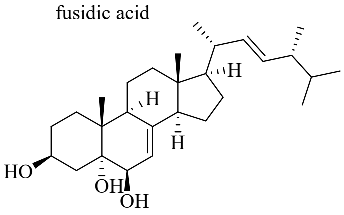

(3β,5α,6β,22*E*,24*R*)-trihydroxy-ergosta-7,22-dien-6-one

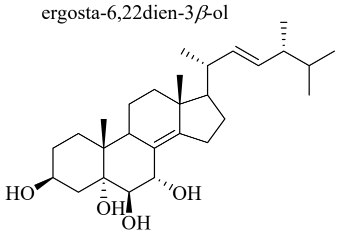

3β,5α,6β,7α-tetrahydroxyergosta-8(14),22-diene

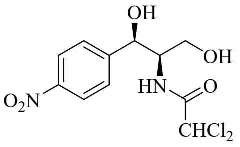

chloramphenicol

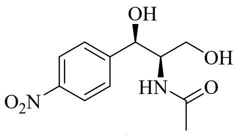

corynecin-I

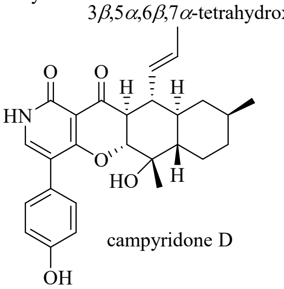

campyridone D

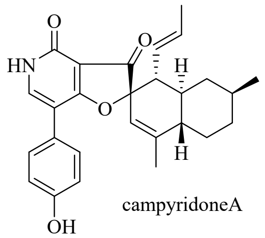

campyridoneA

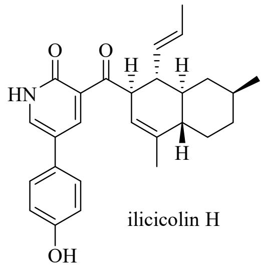

ilicicolin H

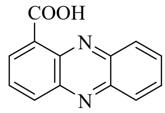

phenazine-1-carboxylic acid

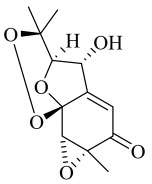

acremine P

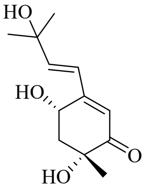

acremine A

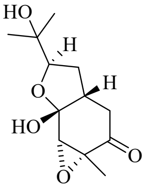

acremine Q
